# Deep-Learning–Based Characterization of Tumor-Infiltrating Lymphocytes in Breast Cancers From Histopathology Images and Multiomics Data

**DOI:** 10.1200/CCI.19.00126

**Published:** 2020-05-26

**Authors:** Zixiao Lu, Siwen Xu, Wei Shao, Yi Wu, Jie Zhang, Zhi Han, Qianjin Feng, Kun Huang

**Affiliations:** ^1^ Guangdong Provincial Key Laboratory of Medical Image Processing, School of Biomedical Engineering, Southern Medical University, Guangzhou, People’s Republic of China; ^2^ Institute of Intelligent System and Bioinformatics, College of Automation, Harbin Engineering University, Harbin, Heilongjiang, People’s Republic of China; ^3^ Department of Medicine, Indiana University School of Medicine, Indianapolis, IN; ^4^ Wormpex AI Research, Bellevue, WA; ^5^ Department of Medical and Molecular Genetics, Indiana University School of Medicine, Indianapolis, IN; ^6^ Regenstrief Institute, Indianapolis, IN

## Abstract

**PURPOSE:**

Tumor-infiltrating lymphocytes (TILs) and their spatial characterizations on whole-slide images (WSIs) of histopathology sections have become crucial in diagnosis, prognosis, and treatment response prediction for different cancers. However, fully automatic assessment of TILs on WSIs currently remains a great challenge because of the heterogeneity and large size of WSIs. We present an automatic pipeline based on a cascade-training U-net to generate high-resolution TIL maps on WSIs.

**METHODS:**

We present global cell-level TIL maps and 43 quantitative TIL spatial image features for 1,000 WSIs of The Cancer Genome Atlas patients with breast cancer. For more specific analysis, all the patients were divided into three subtypes, namely, estrogen receptor (ER)–positive, ER-negative, and triple-negative groups. The associations between TIL scores and gene expression and somatic mutation were examined separately in three breast cancer subtypes. Both univariate and multivariate survival analyses were performed on 43 TIL image features to examine the prognostic value of TIL spatial patterns in different breast cancer subtypes.

**RESULTS:**

The TIL score was in strong association with immune response pathway and genes (eg, programmed death-1 and *CLTA4*). Different breast cancer subtypes showed TIL score in association with mutations from different genes suggesting that different genetic alterations may lead to similar phenotypes. Spatial TIL features that represent density and distribution of TIL clusters were important indicators of the patient outcomes.

**CONCLUSION:**

Our pipeline can facilitate computational pathology-based discovery in cancer immunology and research on immunotherapy. Our analysis results are available for the research community to generate new hypotheses and insights on breast cancer immunology and development.

## INTRODUCTION

The interaction between the tumor and its microenvironment (TME) plays a critical role in cancer development and progression. TME consists of various cells, including fibroblasts and a wide spectrum of immune cells.^1^ The host immune system is crucial in regulating tumor growth by continuous immunosurveillance and initiation of inflammatory reactions.^2,3^ Many observations indicate that tumor-infiltrating lymphocytes (TILs) and their spatial characteristics have significant diagnostic and prognostic values in multiple types of cancers.^4-9^ For example, recent studies suggest that high TIL densities correlate with favorable clinical outcomes in colorectal cancer, non–small-cell lung cancer, and head and neck cancers.^10,11^ Currently, the quantification and scoring of TILs on whole-slide images (WSIs) has been mainly performed and interpreted by domain experts, which can be subjective and influenced by human bias. As a result, automated image analysis methods are desired to reduce labor costs and provide consistent and accurate TIL evaluations. A fully automatic approach for TIL quantification and analysis should involve three technical issues: (1) selection of field of views (FOVs) on WSIs, (2) lymphocyte detection in FOVs, and (3) quantification of TILs for clinical assessment.

**CONTEXT**

**Key Objective**
To develop a fully automatic pipeline that enables accurate quantification and thorough exploration of tumor-infiltrating lymphocytes (TILs) using histopathology image data and multiomics data.
**Knowledge Generated**
Correlations between TIL spatial features derived from our deep-learning–based pipeline and gene expression data indicate that there are different cellular processes associated with the patient’s immune response in triple-negative and other breast cancer subtypes. Both the genomic correlations and survival analysis results imply that the clustering dispersion pattern of TILs is an important factor for evaluating immune response.
**Relevance**
Our image-processing pipeline can be easily used for TIL quantification on histopathology images, and help to reduce labor costs and human bias. The difference between genes in correlation with TIL features in triple-negative and other breast cancer subtypes will bring new insights into future immunologic research for breast cancer treatment.

Many approaches have been proposed for lymphocyte detection.^12-17^ Lymphocytes typically have small (7-10 μm), round, and dark nuclei with little cytoplasm, which is distinctive from malignant (epithelial) cells or stromal cells. Based on these characteristics, some studies first perform nuclei detection algorithms to distinguish all nucleus from cytoplasm in the hematoxylin and eosin (H&E)–stained images, then use a support vector machine algorithm to classify cellular components into different categories based on nuclear morphology.^18,19^ These methods are indirect and may sacrifice the accuracy of lymphocyte detection for overall classification performance. Recently, deep learning has become popular in computer vision and image-processing tasks because of its outstanding performance, and some studies have applied deep-learning methods to detect lymphocytes.^20-22^ However, because these methods require manual preselection of representative tumor regions for each slide, directly applying such models for overall TIL analysis on WSIs remains a practical challenge. Furthermore, this work did not deeply explore the relationships between TILs and omics data.

For instance, Amgad et al^23^ has developed an effective deep-learning–based method for joint region-level and nucleus-level segmentation of TILs, even though their work was limited by the lack of validation on large-scale image datasets and additional analysis between spatial TIL features and biologic data. Saltz et al^24^ have presented global mappings, as well as the spatial organization and molecular correlation of TILs for over 5,000 H&E diagnostic WSIs from The Cancer Genome Atlas (TCGA) dataset, which represented a benchmark for TIL analysis. However, because of the convolutional neural network model in Saltz et al,^24^ with only a calculated lymphocytic probability for each patch, it could not provide more specific cellular information, such as TIL counts and distribution that were related to clinical outcomes. Klauschen et al^25^ summarized different automated TIL scoring approaches in computational pathology and pointed out that it would increasingly contribute to the applications in immune research, but also emphasized that additional omics analysis, including mutational profiling, gene expression, and machine learning, were needed to enhance precision medicine.

Our study extended the limitations of previous work from both the image and biologic analysis perspectives. We first present a fully automatic image-processing pipeline following the clinical steps to quantitatively characterize TILs on histopathologic slide images. Our framework consists of three parts: (1) automatic identification of FOV; (2) a cascade-trained U-net model for lymphocyte detection; and (3) global quantification of TILs on WSIs. We applied our method on 1,000 unannotated TCGA breast cancer diagnostic WSIs and generated TIL maps containing rich cellular information of lymphocytes. We also extracted a set of TIL spatial features based on our TIL maps and explored the relationship between these TIL features and different omics data for different breast cancer subtypes. The associations between TIL features and genetic mutations, as well as gene expression, indicate that there may be different cell activation processes regulating the immune responses in different breast cancer subtypes. A set of features related to the spatial dispersion of TIL clusters was also found to be associated with survival in different breast cancer subtypes. All these analysis results are available for the research community to generate new hypotheses and insights on breast cancer immunology and development. The main contribution of this article is that our work is a complete pipeline using multiple sources, which not only enables fully automatic TIL evaluation on images but also incorporates omics analysis from different biologic data. Our pipeline can be easily extended to histologic images of other cancers and can facilitate the computational pathology-based discovery in immunology and research on immunotherapy.

## METHODS

### Data Source and Selection

Two breast cancer datasets were used in this study, namely, the TCGA-BRCA dataset and the lymphocyte detection dataset released by Janowczyk and Madabhushi.^20^ The TCGA dataset includes matched H&E-stained diagnostic images, transcriptome, somatic mutation, and clinical information for patients with breast cancer. Patients with missing molecular data or images with too-severe cryoartifacts or low-level lymphocyte infiltration were excluded, leaving a set of 1,000 samples. The dataset by Janowczyk and Madabhushi,^20^ denoted as $D_{1}$, includes 200 small images of 200 × 200 pixels at 40× magnification with lymphocyte centers annotated by human experts, which contain a total of 3,064 lymphocytes. The demographic and clinical information of the patients is summarized in Table 1.

**TABLE 1.**
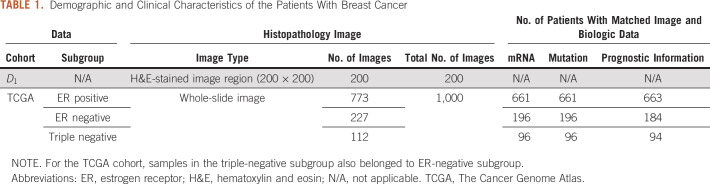
Demographic and Clinical Characteristics of the Patients With Breast Cancer

### Histopathologic Image-Processing Pipeline

**FIG 1.**
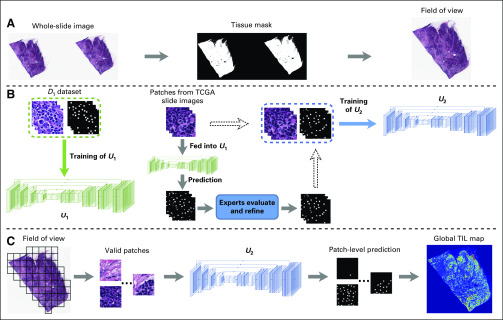
The pipeline for identification of tumor-infiltrating lymphocyte (TIL) maps on whole-slide histopathologic images. (A) Identification of field of view (FOV) for whole-slide image. (B) Cascade training of the U-net model for lymphocyte detection. (C) Generating the global TIL map. TCGA, The Cancer Genome Atlas.

Figure 1A outlines the identification of FOV in our pipeline. Because the H&E stains basic cellular structures either red or pink in an image, we can distinguish different tissues from the background area by color classification. Given a WSI I, we first downsampled I into $I^{\prime }$ by a factor of 16:1. $I^{\prime }$ was then converted from the RGB color space to CIELAB color space. Next, we performed a K-means clustering algorithm to separate the pixels in CIELAB space into three groups. Considering that corners of pathology images are often unstained, pixels in the same cluster as the upper-left corner pixel in $I^{\prime }$ were considered background, whereas the other pixels were considered either tumor or stromal tissues. Denoting the smallest rectangle region containing the largest continuous tissue area in $I^{\prime }$ as $FOV_{I^{\prime }}$, we mapped the coordinates of $FOV_{I^{\prime }}$ onto I and obtained the FOV in the original WSI, $FOV_{I}.$ Finally, $FOV_{I}$ was cropped from I for later processing.

Next, we constructed a U-net–based neural network to identify lymphocytic regions on H&E-stained images. Considering that our annotated lymphocyte dataset might not be large enough to train a robust model from scratch, we adopted the first five blocks of the Resnet18 model^26^ as the encoder in our model. This implementation was inspired by the success of using Resnet18 for objection detection in Ren et al^27^ and Redmon et al,^28^ and we perceived it would help to improve the efficiency and performance of our model. The encoder was then followed by a decoder containing five repeated upsampling blocks, each of which consisted of a concatenation layer, a 2 × 2 deconvolution layer, and two 3 × 3 convolution layers. Parameters in the encoder were initialized with the pretrained weights from He et al,^26^ and the decoder layers were randomly initialized using the Xavier method.^29^

We proposed a cascade-training scheme that involved feedback from domain experts to obtain a robust model for lymphocyte detection. Our framework is illustrated in Figure 1B. In the first stage, $D_{1}$ was used for end-to-end training and evaluation. Note that $D_{1}$ only provided annotations for lymphocyte centers, which did not perfectly match the design of our U-net–based network. Considering that lymphocytes are usually round cells with diameters approximately 8 μm (32 pixels at 40× magnification), we constructed binary lymphocytic masks by dilating each annotated center into a circular area with a diameter of 32 pixels to use the U-net–based model. Eighty percent of $D_{1}$ (160 images of 200 × 200 pixels) were randomly picked out for training, and the remaining 20% were used for testing. Random mirror and random crop were performed to augment the training set. Both the training and evaluation loss converge on $D_{1}$ after training for 200 epochs.

Denoting the U-net model well trained on $D_{1}$ as $U_{1}$, whereas $U_{1}$ can identify lymphocytes on $D_{1}$, it was not robust enough to detect lymphocytes in other cohorts. To further improve the robustness, we performed the second-stage training involving feedback from pathologists on $U_{1}$. In this stage, we used an iterative cycle of review and refinement for training. In the initial iteration, we manually cropped a group of 1,000 patches of 200 × 200 pixels from TCGA WSIs, denoted as $G_{1}$. Fifty percent of $G_{1}$ was collected from immune hotspots with densely clustered lymphocytes, whereas the remaining 50% were randomly collected from tissue regions scattered by lymphocytes. Each patch in $G_{1}$ was fed into $U_{1}$, and the predicted lymphocyte mask was evaluated and refined by two domain experts. Then, we used $G_{1}$ and its refined masks to train $U_{1}$ until convergence. We repeated this iterative process with new groups of patches until the pathologists considered the prediction accuracy to be 0.9. Two iterations were performed on TCGA groups in our experiment. The model after final iteration, denoted as $U_{2}$, was then used to generate TIL maps for all TCGA breast cancer WSIs.

Figure 1C outlines the steps for generating global TIL maps in our pipeline. For each WSI I, its FOV, $FOV_{I}$ was split into nonoverlapping patches of 200 × 200 pixels. Patches with more than 80% background were discarded. The remaining valid patches were then fed into $U_{2}$, and all the patch-level predictions were combined to generate a global TIL map for I. We estimate an overall TIL score TIL% for I by computing the percentage of TIL areas as:

$$TIL\%=\sum_{i}^{K}L_{i}/\sum_{i}^{K}T_{i},$$ (1)

where $L_{i}$ and $T_{i}$ represent the region of lymphocytes and the number of tissue pixels in the *i*th valid patch in I, respectively, and K represents the total number of valid patches in I.

### Spatial Features of TIL

We estimated 42 TIL spatial features based on the TIL maps derived from our pipeline. For each TIL map, we first picked out TIL patches as independent data points. Then, we used the *APCluster* R package^30^ to obtain local TIL cluster patterns by applying the affinity propagation algorithm^31^ on the data points. Next, we used the *clusterCrit* R package to extract statistical TIL spatial features from the TIL clusters, as listed in Table S1 of the Data Supplement.

### Analysis of the Relationship Between TIL Score and Multiomics Data, Including Gene Transcription and Somatic Mutations

We divided all patients in the TCGA-BRCA cohort into three subtypes, estrogen receptor (ER) positive, ER negative, and triple negative, based on patients’ status of markers, including ER, progesterone receptor, and human epidermal growth factor receptor 2. We first computed and sorted the Spearman correlation coefficients between gene transcription levels and the TIL score, as well as all the 42 TIL features derived from imaging data for each subtype. Then we select the genes whose transcription levels were significantly correlated with TIL scores (*P*_Spearman_ > .3) for each subtype. For the selected gene symbols, we performed function and pathway enrichment analysis using Ingenuity Pathway Analysis (IPA).

In addition, for each gene with somatic mutation information available, we performed the Wilcoxon rank sum test between patients with and without nonsynonymous mutations for the TIL scores in each subtype. Specifically, we selected genes with nonsynonymous mutations in at least 10 patients. In total, we had 33 genes for the ER-positive patients, 61 genes for the ER-negative patients, and 25 genes for the triple-negative group.

### Machine-Learning Methods for Prognostic Prediction

We performed both univariate and multivariate survival analysis using the Cox proportional hazard model for patients with breast cancer of different subtypes, based on the TIL spatial statistical features. For the univariate method, we followed the approach in Uhlen et al.^32^ Specifically, for each feature, we selected different cutoffs (from the 20 percentile to the 80 percentile, with 1 percentile increments) to stratify the patients into two groups using the approach in Uhlen et al.^32^ Different cutoffs led to different choices of patient groups. According to the group information, we then calculated the *P* values of the log-rank test for comparing survival times between the two groups.

For the multivariate method, we compared the prognostic power of different approaches by stratifying patients with cancer into two subgroups (ie, the high- and low-survival risk groups) with different predicted outcomes. Specifically, for all the TIL spatial features of different patients, the K-means clustering algorithm was adopted to aggregate the patients into different subgroups. Then, we tested whether these groups had significantly different survival outcomes using the log-rank test. Finally, based on the divided groups of different patients, the Lasso-based feature selection model was applied to identify important biomarkers that can distinguish different patient groups.

## RESULTS

### Detection and Quantification of TIL Spatial Patterns in Histopathologic Images

**FIG 2.**
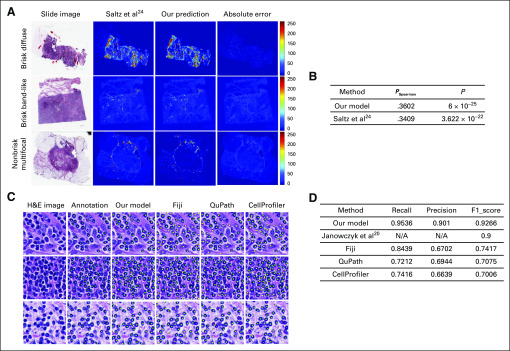
Evaluation and visualization of results for tumor-infiltrating lymphocyte (TIL) detection. (A) TIL maps for three breast cancer whole-slide images (WSIs) from The Cancer Genome Atlas in different TIL patterns; (left) hematoxylin and eosin (H&E) whole-slide diagnostic image; (middle) predicted TIL maps; (right) absolute error between our map and the map from Saltz et al.^24^ Values in the color bar represent different TIL levels: 0 (blue) corresponds to patches without TIL, and 250 (red) corresponds to patches filled with TILs. (B) Spearman correlation for comparison of TIL proportion from WSI TIL maps and molecular estimates of TIL content from genomics assays.^36^ (C) Cell-level results of lymphocyte detection predicted by our model and the three software programs. Green, yellow, and magenta dots in the prediction represent true positive, false positive, and false negative, respectively. (D) Performance comparison between our model and the three software programs for lymphocyte detection.

To evaluate the effectiveness of the proposed U-net for lymphocyte detection, we tested and compared our model with three widely used open-source biomedical image analysis software programs: CellProfiler,^33^ QuPath,^34^ and Fiji,^35^ on $D_{1}$. Comparison of lymphocyte detection results with CellProfiler, QuPath, Fiji, and U-net are provided in Figure 2C and Fig 2D. From Figures 2C and 2D, we observe that Fiji achieves the highest average recall (0.8439) and F1-score (0.7471) among the three existing software programs. However, the proposed U-net model had substantially better performance (recall, 0.9536; precision, 0.901; F1-score, 0.9266). This makes sense because the existing software programs perform unsupervised algorithms for nuclei detection, whereas our U-net model was specifically trained for lymphocyte detection.

We applied our pipeline for identification of TIL maps to 1,000 TCGA-BRCA WSIs. Because there was no available TIL annotation for TCGA WSIs, we compared our results with the only referable TIL maps in Saltz et al.^24^ Figure 2A shows the visualization of TIL maps for three TCGA WSIs in different TIL patterns: Brisk Diffuse pattern, showing a strong immune infiltration within the tumor; Brisk Band-like pattern, showing immune infiltration forming boundaries bordering the tumor; and Nonbrisk Multifocal pattern, showing a weak immune response with loosely scattered TILs. Figure 2A demonstrates that our framework can efficiently identify different types of immune spots.

We also performed a more specific evaluation for the cell-level performance of our framework compared with the TIL maps in Salz et al.^24^ We compared the TIL scores derived from our method with the molecular estimates of TIL content from genomics assays.^36^ The Spearman correlation coefficients between TIL proportion from imaging and molecular estimates are shown in Figure 2B. As can be seen, the TIL maps generated by our method are more consistent with the molecular estimates (*P*_Spearman_, .3602; *P* = 6 × 10^–25^) than TIL maps in Salz et al^24^ (*P*_Spearman,_ .3409; *P* = 3.622 × 10^–22^). This improvement indicates that our cell-level TIL maps are not only in line with the associated molecular data, but also provide more accurate and detailed immune estimation than patch-level TIL maps.

### Correlate TIL Spatial Features With Gene Expression

**FIG 3.**
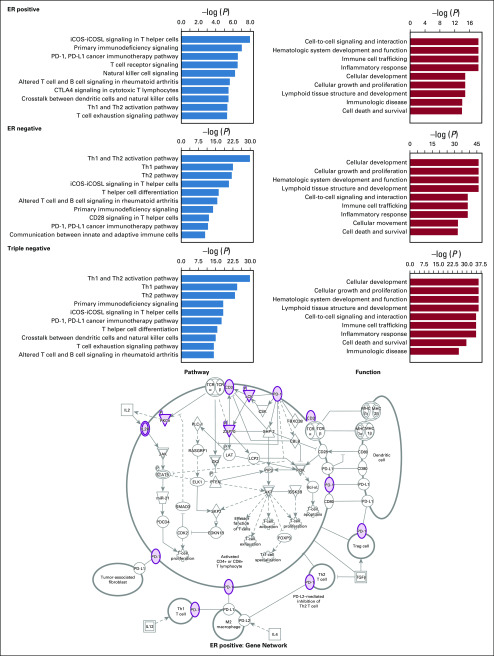
Enrichment of different canonical pathways and functions in three breast cancer subtypes for genes whose transcription levels showed high correlations with tumor-infiltrating lymphocyte (TIL) score. PD-1, programmed death-1; PD-L1, programmed death-ligand 1.

To investigate which genes contribute to the development of immune infiltrating in different breast cancer subtypes, we performed enrichment analysis for the genes that were in high correlation with TIL scores. The enrichment results are shown in Figure 3, with the gene lists in Table S2 of the Data Supplement. When we applied a threshold value of 0.3 to the Spearman correlation coefficients, 54, 307, and 263, genes were selected for ER-positive, ER-negative, and triple-negative breast cancer subtypes, respectively. Among them, many were immune response–related genes. For instance, important immune therapy–related genes, such as *PDCD1* (also known as programmed death-1 [PD-1] and CD279) and *CTLA4* were both observed in all three subtypes. As can be seen in Figure 3, enrichment analysis on canonical pathways and functions from IPA confirmed the strong and consistent enrichment in processes related to immune and inflammatory responses in all three subtypes. An example of the strong involvement of the cancer immunotherapy pathway is also shown with genes observed for the ER-positive patient, marked in gray with magenta boundaries in Figure 3 (ER positive: Gene Network).

We also performed Spearman correlation between the TIL spatial features and the gene expression data. We found that in both the ER-positive and ER-negative subtypes, the “banfeld_raftery” feature, which represents the TIL cluster extent, was highly correlated with immune therapy genes, such as *CD38* and *CXCL9*. These genes are also correlated with the TIL score. But for the triple-negative subtype, we found that there were high correlations between the “g_plus” feature and genes (*PCDHGA2*, *PCDHGA3*, and *PCDHB12*) that have unusual immunoglobulin-like organization similar to that of B-cell and T-cell receptor gene clusters. These genes have not been observed to be associated with TIL score. Because the “g_plus” feature represents the cluster dispersion, these observations may imply that the TIL area and TIL distribution in the triple-negative subtype are regulated by different sets of genes leading to different immune response.

### Compare TIL Score With Somatic Mutation Status of Genes

**FIG 4.**
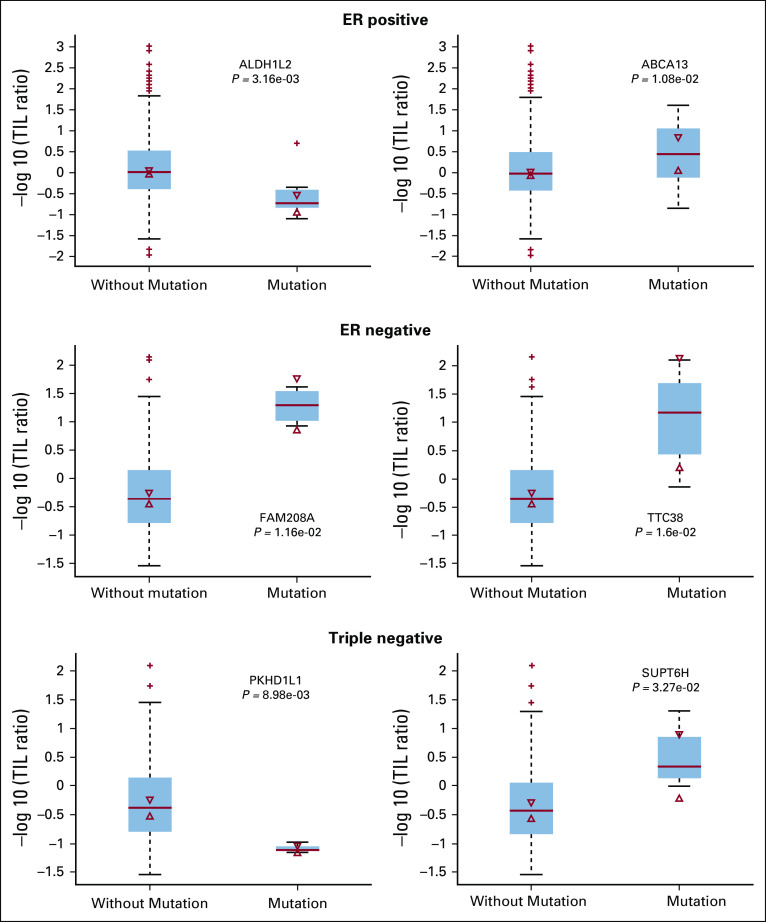
Examples of the associations between tumor-infiltrating lymphocyte (TIL) score and somatic mutations. ER, estrogen receptor.

We also investigated the relationship between somatic mutations and TIL scores. Specifically, for each gene, the patients with and without somatic mutations were separated into two groups, and the rank sum test was applied to test whether there was any difference between the TIL scores. Figure 4 demonstrates two representative examples of the results for each breast cancer subtype. Table S3 in the Data Supplement lists the *P* values for genes whose somatic mutation status is significantly associated with the TIL score (*P* < .05). It is interesting to observe that the genes whose mutation status was strongly associated with the TIL scores were different between the ER-positive and ER-negative groups, whereas there were some similarities between the ER-negative and triple-negative breast cancer groups.

### TIL Spatial Pattern Predicts Survival in Different Breast Cancer Subtypes

**FIG 5.**
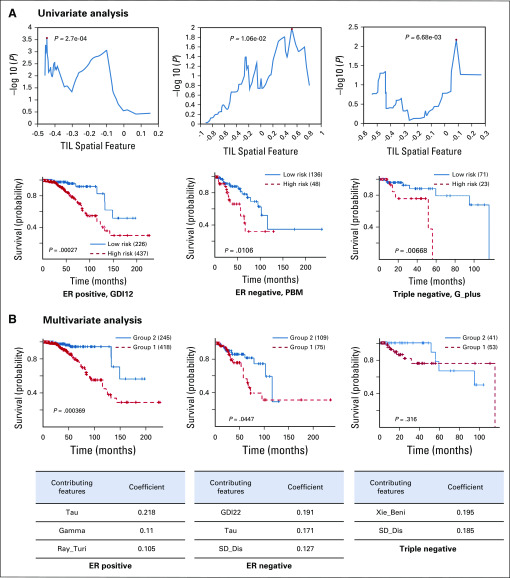
Associations of tumor-infiltrating lymphocyte (TIL) spatial features with survival outcomes in different breast cancer subtypes. (A) Univariate analysis of the prognostic value for single TIL spatial features in different subtypes. (B) Multivariate analysis of the prognostic value for all the TIL spatial features in different subtypes; (top) represents the Kaplan-Meier curve by multiple TIL features; (bottom) represents the main contributing TIL features and their coefficients for survival prediction.

We also examined whether the spatial patterns of TIL might affect or be associated with patient survival in different breast cancer subtypes using both univariate and multivariate methods. We calculated 43 spatial statistical features for the detected TILs for each patient. Results of survival-associated TIL features are shown in Figure 5. For univariate analysis, we adopted the approach in Uhlen et al,^32^ where multiple partitions of the patient cohort based on different thresholds were used for log-rank tests, and the threshold leading to the most significant difference was selected for the specific spatial feature. For multivariate analysis, a Lasso-Cox regression model was used to select the features, with large weights for influencing patient survival times. It can be observed that different subtypes tend to be separated by different TIL spatial features in both univariate and multivariate analysis. In addition, the univariate results are better than the multivariate results. This unintuitive result is due to the fact that the multivariate uses the Lasso-Cox model with a single cutoff for separating the patients, whereas the univariate used multiple cutoff values to identify the best separation.

## DISCUSSION

In this article, we proposed an automatic pipeline based on a cascade-training U-net model to generate high-resolution TIL maps on WSIs. Both qualitative and quantitative results demonstrate that our framework can provide reliable quantification of TILs from H&E-stained histopathology images. The global TIL maps we present can also be used for in-depth biologic analysis.^37-39^ For example, TILs in different tissue regions can be examined separately for survival prediction.^40,41^

The high-resolution TIL maps then enable comprehensive integrative analysis with multiomics data and clinical outcomes. The correlation analysis between the TIL score and gene transcription levels clearly confirmed the validity of the quantification of TILs using our method based on the strong enrichment of immune response genes and pathways. In particular, the strong association with immunotherapy pathway and genes (eg, PD-1 and *CLTA4*) suggests that the WSI-based analysis can be potentially used in the future for assessing or predicting immunotherapy potential.

The comparative analysis for genes with somatic mutations suggests that multiple genes affect the TIL score. However, because the somatic mutations are genetic alterations in the tumor (epithelial) cells, their effects on the TIL score are indirect through the interaction within the TME. The mechanisms for the association warrant additional investigation. In addition, the observation that different subtypes of breast cancers show TIL associations with mutations from different genes suggests that different genetic alterations may lead to similar phenotypes. For the triple-negative subtype, observations between TIL spatial features and gene expression data show that there may be different cell activation processes governing the patient’s immune response.

Last but not least, we also demonstrated that different spatial statistical features for the TIL distributions can be potentially used to predict patient outcomes (ie, survival times). Most of these spatial features (eg, GDI12, PBM, Tau, Gamma) were associated with dispersion and extent of the clustering, suggesting the clumping patterns of the TILs are important indicators of patient outcomes. Thus, except from the overall TIL infiltration, clustering of dispersion of TILs on histopathologic images should receive more attention in clinical settings and will be of great interest as a future research direction.

Overall, in this study, we developed an effective deep-learning–based pipeline for detecting TILs at the cellular level from WSIs of breast cancers. This pipeline was validated on multiple datasets, and correlation analysis with molecular data confirmed its strong associations with immune responses. Our results provide a resource for breast cancer biologists and informaticians for deep investigations between TIL patterns and molecular and clinical data, which will lead to new hypotheses and insights for both cancer biology and translational biomarker discovery. This pipeline can also be applied to study other types of cancers.
